# Tracheal Intubation in Emergency Departments in China: A National Cross-Sectional Survey

**DOI:** 10.3389/fmed.2022.813833

**Published:** 2022-02-25

**Authors:** Yili Dai, Joseph Harold Walline, Heng Yu, Huadong Zhu, Jun Xu, Xuezhong Yu

**Affiliations:** ^1^State Key Laboratory of Complex Severe and Rare Diseases, Department of Emergency, Peking Union Medical College Hospital, Chinese Academy of Medical Science and Peking Union Medical College, Beijing, China; ^2^Accident and Emergency Medicine Academic Unit, Prince of Wales Hospital, Chinese University of Hong Kong, Hong Kong, Hong Kong SAR, China; ^3^Department of Emergency, Shapingba District People's Hospital, Chongqing, China

**Keywords:** China, cross-sectional studies, emergency department, tracheal intubation, rapid sequence intubation

## Abstract

**Background:**

Tracheal intubation is a necessary but risky procedure performed in emergency departments (EDs) around the world. Relatively high morbidity has been encountered in Chinese EDs, which has raised concerns about peri-intubation ED management. This study aimed to investigate intubation procedures and identify any areas for improvement in Chinese EDs.

**Methods:**

This was a questionnaire-based survey lasting 1 month (March 2021) in 41 tertiary-care hospital EDs in mainland China. The primary outcome was complications associated with intubation. Secondary outcomes were the first-pass success rate and blood pressure variations during intubation. Univariate and binary logistic regression analyses were used to find possible risk factors for first-pass intubation failure.

**Results:**

In total, 1,020 replies were analyzed out of 1,080 surveys submitted (94.4% response rate). Most patients were elderly men with severe medical conditions like cardiac arrest (24.8%). In total, 97.2% of patients were given preoxygenation, and 48.1% received some form of pretreatment. Induction drugs (e.g., etomidate and ketamine) were less often used: 39.9% of intubations used sedatives, 5.5% used analgesics, and only 5.3% used muscle relaxants. The overall first-pass intubation success rate was 85.7% and was accompanied by a 19.8% adverse event rate. A marked decrease in blood pressure after intubation was also identified.

**Conclusion:**

This survey found an 85.7% tracheal intubation first-pass success rate (which is relatively high compared to other countries) and a 19.8% adverse event rate (which is also relatively high). Given the very low rate of using induction medications (5.3% used muscle relaxants), future education should focus on induction drugs and traditional intubation techniques.

## Introduction

Tracheal intubation is an essential and frequently used skill in the emergency department (ED). Most patients presenting to the ED with cardiac arrest, respiratory failure, or other serious clinical situations require intubation. Statistically, ED physicians handle up to 81% of intubations by themselves (1). Intubation is, therefore, a key skill for any ED physician.

Emergency department intubation is also a potentially risky procedure, carrying a morbidity rate of 12% according to a recent study in the USA (2). Morbidity from intubation in EDs in the UK was greater than in operating rooms (ORs) according to two recent studies (3, 4). This may be because many ED patients requiring intubation are complicated, with severe cardiopulmonary and infectious diseases, which may increase the incidence of hypotension or hypoxia during intubation in the ED. Given the emergent nature of these cases, there is often insufficient time to ensure proper patient fasting, to thoroughly screen patients' past medical records, or to thoroughly prepare all tools for intubation.

Recognizing the status of intubation management in a country is vital to identify deficiencies and correct any shortcomings. However, few national-scale investigations in China have attempted to explore the characteristics of ED intubations. This study aimed to illuminate the existing circumstances of ED-based tracheal intubations in China. By elucidating the common peri-intubation ED management choices, we hope to identify any problems and provide potential corrective recommendations to reduce future rates of ED intubation adverse events in Chinese EDs.

## Methods

### Study Design and Setting

This was a national cross-sectional survey. We selected tertiary-care hospitals according to China's geographical distribution. The Chinese mainland, according to current government statistical reporting, is divided into seven regions. At least one province in each region was chosen to participate in the study. Ultimately, 41 “Class A-III” hospitals (the highest grade of Chinese hospitals, which are urban, tertiary, and teaching hospitals) from 19 provinces participated in this trial. This study was registered in China (ChiCTR2100043745) and received ethics approval on December 22, 2020.

### Selection of Participants

Adult patients who had a tracheal intubation procedure attempted in an enrolled Chinese ED met inclusion criteria. Other cases of intubation, such as being intubated in the field or in a non-ED part of the hospital, or those patients with data missing were excluded. Patients underwent routine treatment as per the local hospital's protocol and each patient's attending physician's orders. No additional interventions were recommended or required in this study protocol.

This study lasted for a month, from March 1, 2021, to March 31, 2021. March was chosen as it avoids both spring and summer temperature extremes and is free of major national holidays. During the study period, investigators in each enrolled ED entered study data on an internet-based survey form. This form included patient characteristics, intubation conditions, and peri-intubation management decisions (e.g., any pretreatment or preoxygenation methods, induction medication, and intubation devices used). Investigators filled out the survey either on a paper printout or through a web page linked to a centralized Research Electronic Data Capture (REDCap) database. REDCap is a secure web-based software platform designed to support data capture for research studies, providing (1) an intuitive interface for validated data capture; (2) audit trails for tracking data manipulation and export procedures; (3) automated export procedures for seamless data downloads to common statistical packages; and (4) procedures for data integration and interoperability with external sources (5). Every hospital appointed a physician to oversee all data entries. There were random checks every 2 weeks to ensure data accuracy. Before analyzing study data, each questionnaire entry was rechecked for repetition, errors, or incomplete areas.

The primary outcome measure was the presence of any adverse events. Adverse events could include desaturation (defined as a SpO2 <90%), hypotension [defined as systolic blood pressure (SBP) <90 mmHg], aspiration, airway trauma, cardiac arrest, or death during the intubation procedure in the ED. Secondary outcomes included the rate of first-pass intubation success and blood pressure variations during intubation.

According to existing research data, the incidence of adverse events during ED intubation has been documented to be about 12%. With an allowable error of 2%, α = 0.05, 1–β = 0.8, and a CI of 95%, the target sample size for this study was calculated by Power Analysis & Sample Size (PASS) software (NCSS Incorporation, Kaysville, Utah, USA) to be 1063.

We downloaded the raw data from the REDCap database and analyzed it utilizing Statistical Product and Service Solutions (SPSS) version 22 (IBM Incorporation, Armonk, New York, USA), GraphPad Prism 8 (GraphPad Software Incorporation, San Diego, California, USA), and Python 3 (Python Software Foundation, Beaverton, Oregon, USA). Continuous variables were presented as medians with quartiles, and categorical variables as frequencies with percentages. Wilcoxon signed-rank tests were used to compare blood pressure changes before and after intubation. Chi-square tests were used to compare categorical variables. Univariate and binary logistic regression analyses were conducted to find possible factors influencing the success of first-pass intubation. First-pass intubation success was defined as the dependent variable, and patients' underlying disease, sex, age group, intubator experience, intubator professional rank, presence of an emergent condition, intubation devices, or induction drugs were screened for the possible association. Items with a *p* < 0.1 in the univariate analysis or significant clinical significance were entered into multivariate analysis.

## Results

This study collected a total of 1,080 questionnaires. After screening, 14 incomplete, 25 beyond the study period, eight pediatric, seven duplicate, and six questionnaires submitted in error were excluded, leaving a total of 1,020 questionnaires for statistical analysis (shown in Supplementary Figure 1).

**Figure 1:**
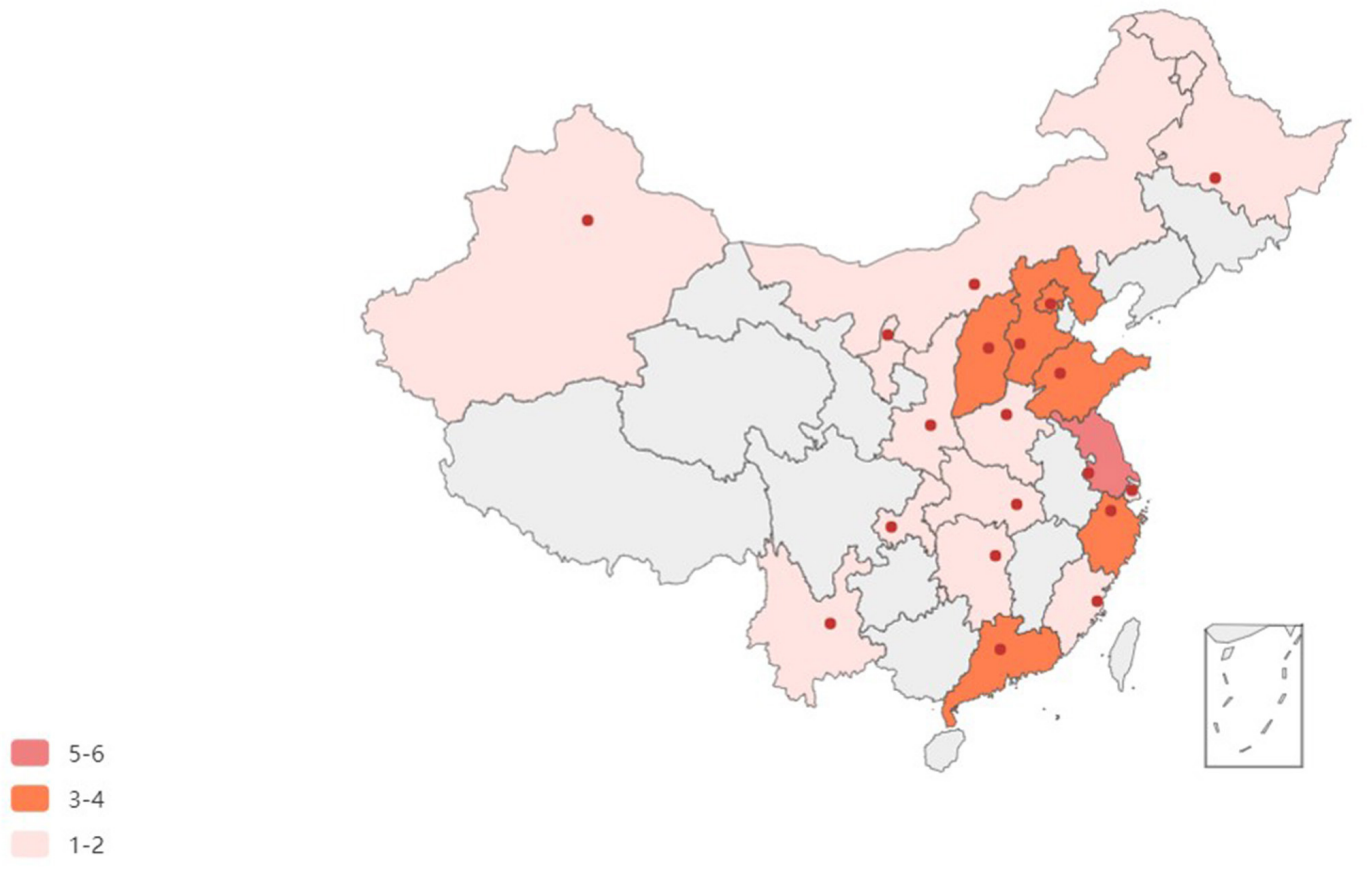
Nationwide map of enrolled hospitals.

**Figure 2:**
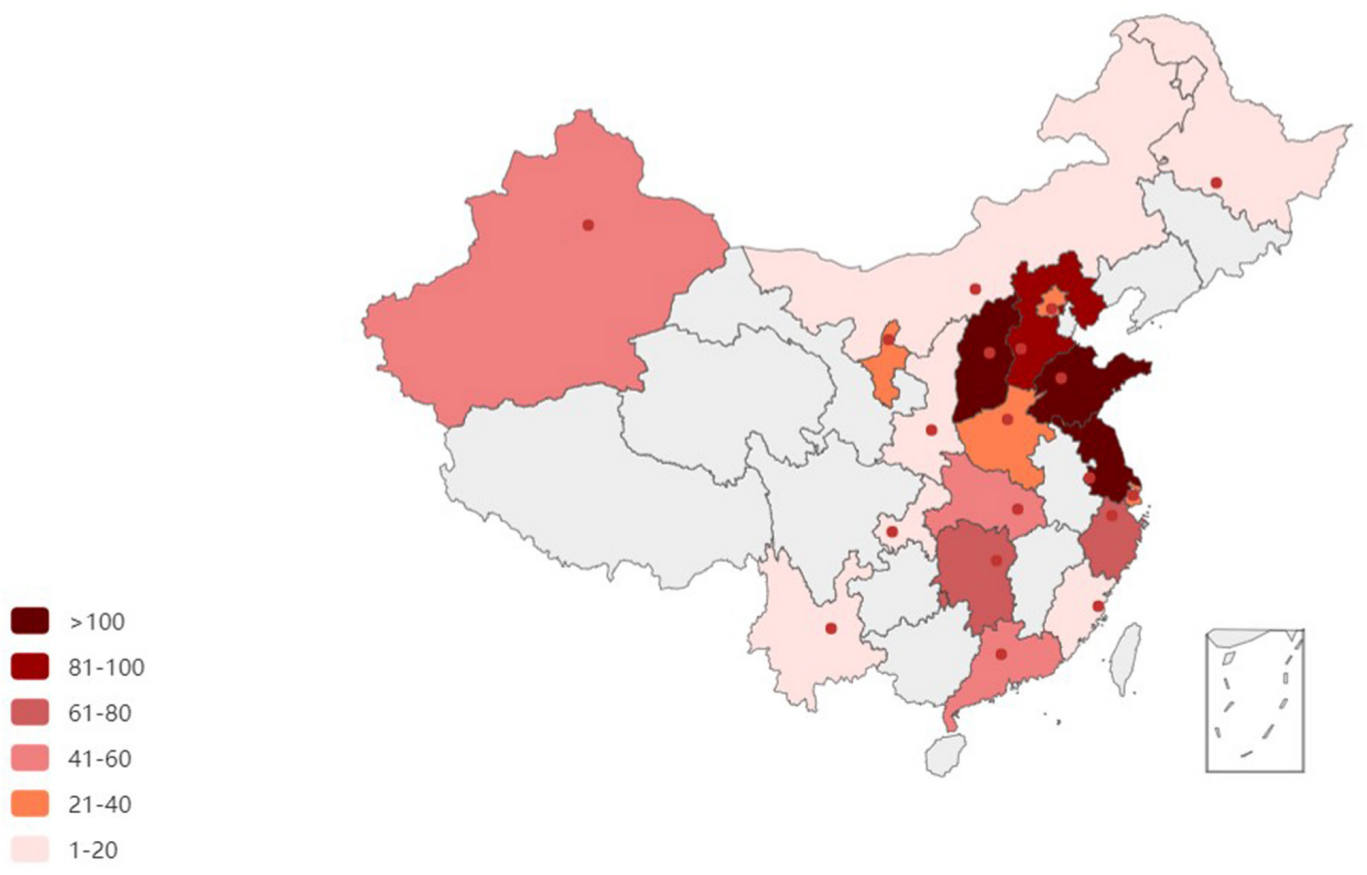
Nationwide map of case distribution.

There were 41 enrolled hospitals in this study. At least one representative hospital from more than half the provinces (19 out of 34, 55.9%) in China was enrolled in this trial. More enrolled hospitals were in the more populous eastern coastal cities, with four or more hospitals located in Guangdong, Jiangsu, and Shanxi provinces (shown in Figure 1). The distribution of intubation frequency based on area is shown in Figure 2.

**Figure 3:**
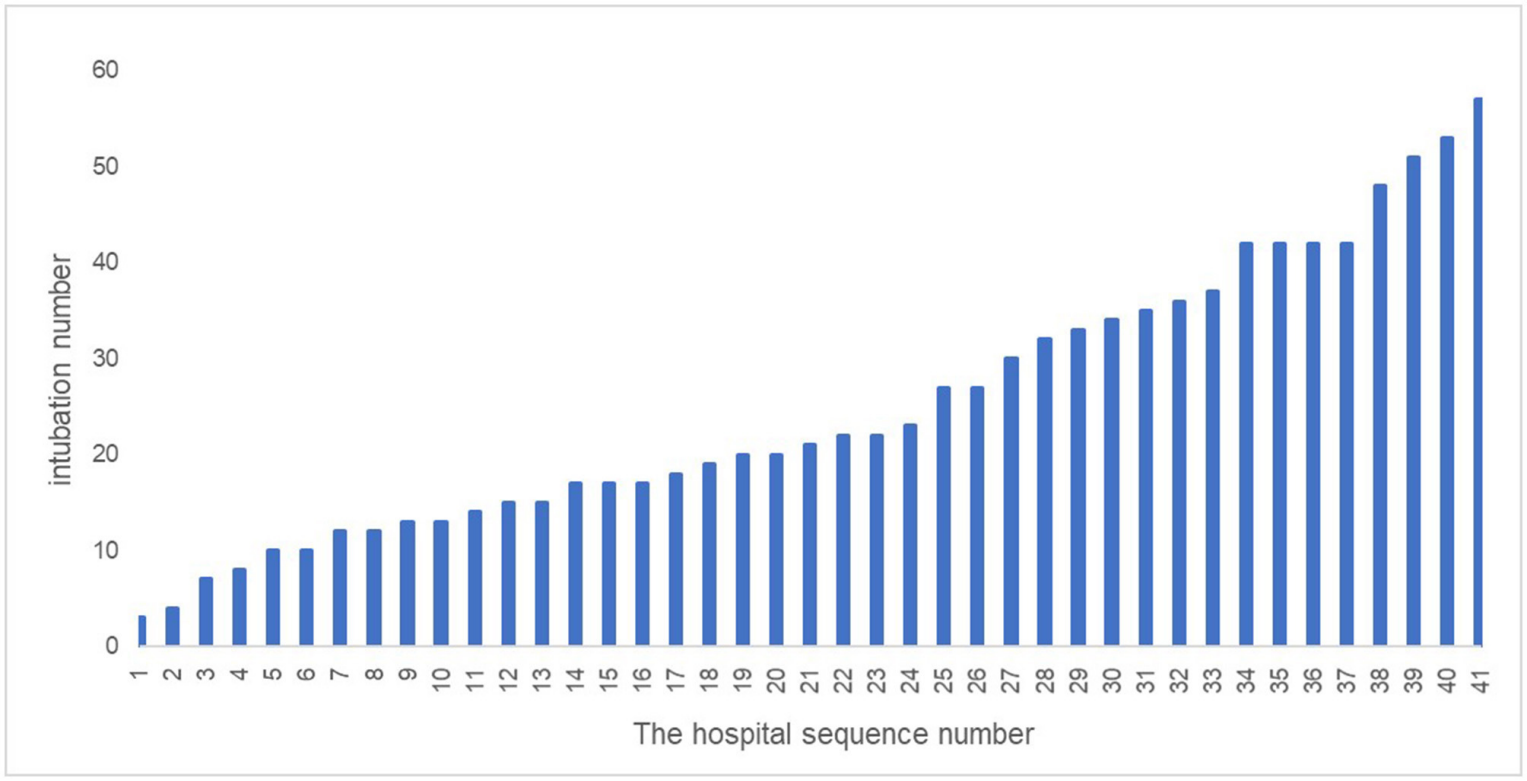
Emergency department (ED) intubation frequency in each hospital in March 2021.

The number of ED intubations varied between different hospitals, even in the same province. The median was 21 intubations [interquartile range (IQR), 13.5–35.5]. Figure 3 illustrates the total intubation numbers by the enrolled hospital.

In total, 953 intubations were performed by ED physicians (93.4%), 67 by anesthesiologists (6.3%), and 65 (6.4%) by nurses in the ED. The performing physicians were either attending physicians in 457 (44.8%) or resident physicians in 354 (34.7%) of the cases. In total, 643 (63.0%) reported being skilled in intubation (number of prior intubations >50). Intubations were slightly more likely to occur during off-hours between 5 pm and 8 am in 544 patients (53.3%) (Table 1).

**Table 1:** General characteristics of ED intubations.

**Characteristics**	***N*** **(%)**
Intubation department	
Emergency department	953 (93.4%)
Anesthesiology department	67 (6.6%)
Intubation time	
On-hours (8 am−5 pm)	476 (46.7%)
Off-hours	544 (53.3%)
Intubation experience	
>50	643 (63.0%)
≤ 50	377 (37.0%)
Qualification	
Associate doctor and above	137 (13.5%)
Attending doctor	457 (44.8%)
Resident doctor	354 (34.7%)
Intern	7 (0.7%)
Nurse	65 (6.4%)

In total, 685 patients (67.2%) who underwent intubation were male. The median age was 66 years, and 473 of patients (46.4%) were aged between 60 and 80. The causes for intubation were respiratory failure in 209 patients (20.5%), circulatory failure in 191 patients (18.7%), central nervous system disease in 362 patients (35.5%), cardiac arrest in 253 patients (24.8%), and “others” in five patients (0.5%). In addition, 797 (78.1%) subjects were intubated immediately on ED arrival (Table 2).

**Table 2:** Characteristics of enrolled ED intubation patients.

**Characteristics**	***N*** **(%)**
Sex	
Male	685 (67.2%)
Female	335 (32.8%)
Age	
18–40	84 (8.2%)
40–60	305 (29.9)
60–80	473 (46.4%)
80–100	158 (15.5)
Cause	
Respiratory failure	209 (20.5%)
Circulatory failure	191 (18.7%)
CNS disease	362 (35.5%)
Cardiopulmonary arrest	253 (24.8%)
Others	5 (0.5%)
Emergency intubation	797 (78.1%)

Looking at pre-intubation medications or fluid resuscitation, 631 patients (61.9%) were not pretreated at all, while 314 patients (30.8%) received vasoactive agents, and 179 patients (17.5%) received intravenous (IV) fluid resuscitation. In contrast, oxygenation-improving techniques (such as preoxygenation, denitrogenation, or apneic oxygenation) were much more common. Only 28 patients (2.7%) were not pre-oxygenated before intubation. Oxygenation-improving methods were nasal cannula in 330 patients (32.4%), oxygen masks in 206 patients (20.2%, including venturi and non-rebreather masks), noninvasive positive-pressure ventilation in 99 patients (9.7%), and high-flow oxygen delivery methods in 73 patients (7.2%). Bag-valve-mask ventilation was performed in 542 patients (53.1%).

Turning now to the intubation procedure itself, Table 3 displays the variety of induction agents used for intubation in the ED. Less than half of the patients were given sedatives before intubation. The most popular drugs were propofol [used in 206 cases (20.2%)] and midazolam [used in 177 patients (17.4%)]. ED physicians rarely used analgesics before intubation, but when they did, they used IV fentanyl [in 42 patients (4.1%)]. In terms of muscle relaxation, scarcely any patients were paralyzed. For those who were, non-depolarizing muscle relaxants were used in 49 (4.8%) and depolarizing muscle relaxants in 5 (0.5%). Furthermore, compared with anesthesiologists (65.7%), the proportion using sedatives was significantly lower in emergency physicians (38.1%) (*p* < 0.01). As for muscle relaxants, anesthesiologists (10.4%) were about two times as likely to use these agents compared to emergency physicians (4.9%) (*p* = 0.051). In addition, fewer sedatives were utilized among patients intubated immediately on arrival (31.2 vs. 70.9%, *p* < 0.01).

**Table 3:** Induction agents used during ED intubations.

		***N***	**Percent**
Sedation			
	No sedative	613	60.1
	Midazolam	177	17.4
	Propofol	206	20.2
	Etomidate	22	2.2
	Ketamine	0	0
	Others	30	2.9
Analgesia			
	No analgesic	964	94.5
	Fentanyl	42	4.1
	Morphine	4	0.4
	Others	10	1.0
Muscle relaxants		
	No paralytic	967	94.7
	Rocuronium	39	3.8
	Succinylcholine	5	0.5
	Shunatracurium	8	0.8
	Others	2	0.2

With the steady worldwide increase in intubation equipment, Chinese ED physicians now have a plethora of options, from the traditional direct laryngoscope to a variety of direct and indirect visualization equipment. In this study, 819 intubations (80.3%) were performed using a video laryngoscope, greatly exceeding traditional direct laryngoscopy, which was still used in 192 cases (18.8%).

Looking at first pass success rates and remedies after failure, initial intubations were successful in 874 (85.7%) patients, while 125 (12.3%) required a second attempt, and 20 (2.0%) needed three attempts. In one patient (0.1%), attempts were finally given up as futile. When ED physicians faced first-pass intubation failure, the most common alternative strategy was to change to a more experienced operator, which accounted for 41.1% (60 of 146 first-pass failures) and 14.4% (21 of 146) opted to try again personally, and 10.3% (15 of 146) selected another intubation device. No supraglottic airway devices were used and no cricothyroidotomy procedures were performed.

**Figure 4:**
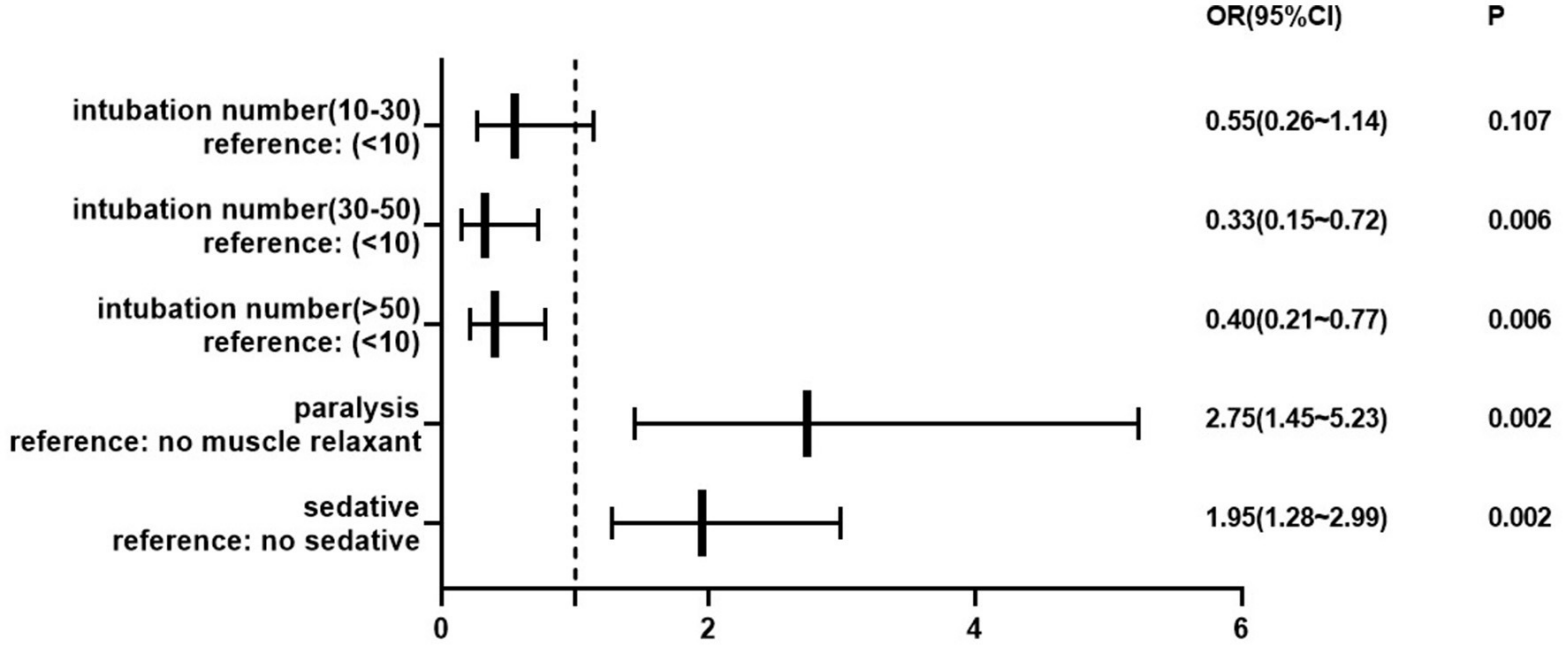
Forest map of the risk of first-pass intubation attempt failure. The dotted line demarcates protective factors on the left and risk factors on the right. *P* < 0.05 represented statistically significant.

Three aspects were considered in univariate regression screening for risk factors associated with initial intubation failure. From patient characteristics, sex (*p* = 0.452), age groups (*p* = 0.295), and concurrent diseases (*p* = 0.005) were included. The intubator's home department (*p* = 0.831), their professional rank (*p* = 0.231), and number of prior intubations (*p* = 0.007) were analyzed. Regarding intubation conditions, emergency intubation (*p* = 0.127), additional intubation devices utilized (*p* = 0.643), sedatives (*p* < 0.001), analgesia (*p* = 0.138), and muscle relaxants (*p* = 0.001) were considered. Unsurprisingly, there was an association between the professional rank of intubators and prior intubation numbers. Finally, the binary logistic model analyzed age, concurrent diseases, intubator department, prior intubation numbers, emergency intubation, devices, and three types of induction drugs. Hosmer and Lemeshow's test showed a good fit (*p* = 0.462). Results suggested that prior intubation numbers, the use of sedative agents, and the use of muscle relaxants were associated with first-pass success or failure rate. The more intubation once performed, the higher the success rate. However, using sedatives and muscle relaxants may increase the risks (see Figure 4).

**Figure 5:**
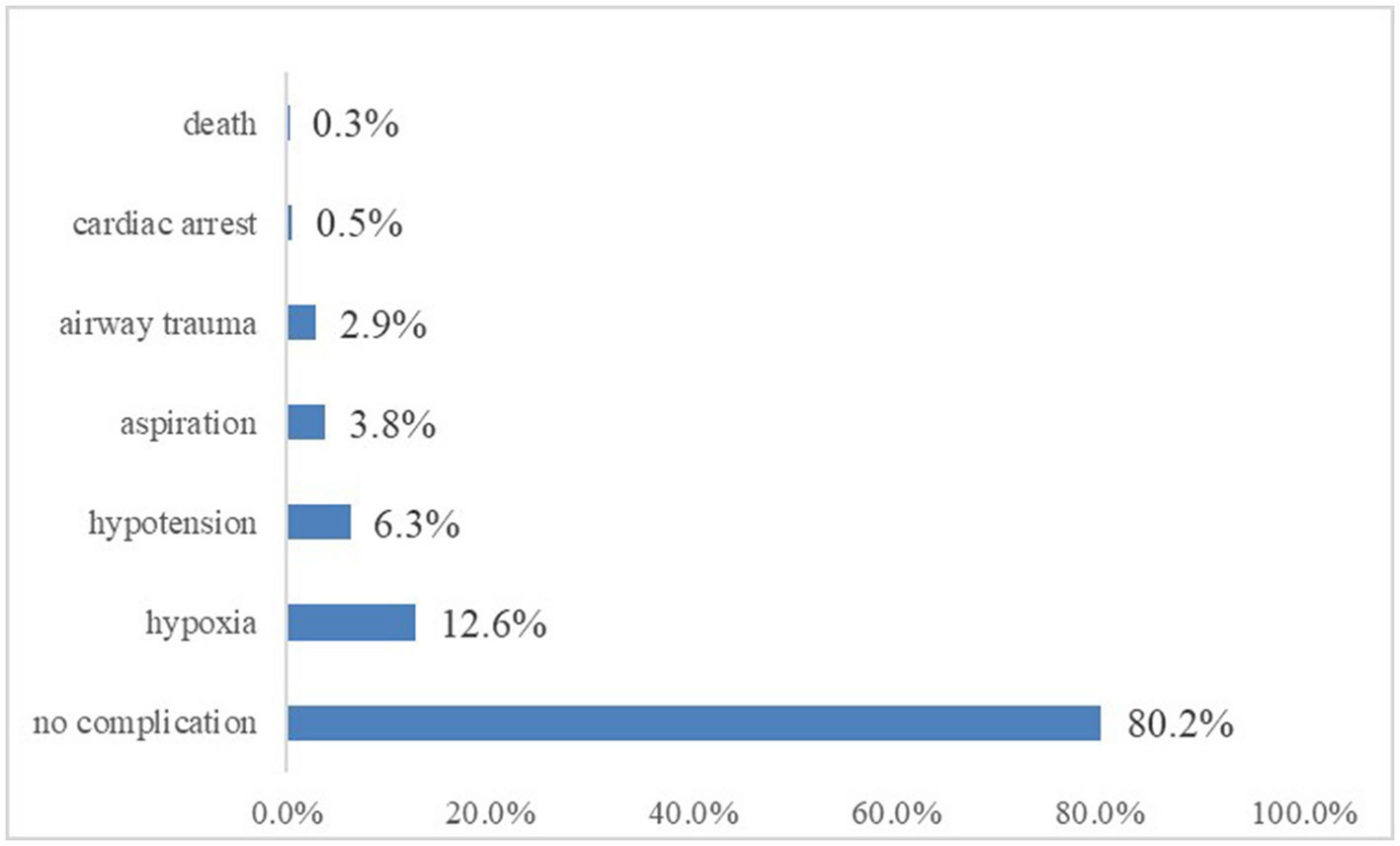
Incidence of different complications during ED intubation.

Several kinds of complications may occur during tracheal intubation, especially in patients with serious underlying clinical conditions. Excluding patients with cardiac arrest, the remaining 767 patients experienced a variety of adverse events, including hypoxia in 97 cases (12.6%), hypotension in 48 (6.3%), aspiration in 29 (3.8%), and airway trauma in 22 (2.9%). Six patients suffered severe adverse events such as cardiac arrest or death (see Figure 5).

Among all subjects, blood pressure was detectable in 654 of 1,020 cases (64.1%). The median SBP was 130 mmHg before intubation and 121.5 mmHg after intubation. The median diastolic blood pressure (DAP) was 77 mmHg before and 72 mmHg after. The median mean arterial pressure (MAP) was 94.8 mmHg before and 88.8 mmHg after intubation. Wilcoxon signed-rank tests showed significant differences in blood pressure before and after the intubation (*p* < 0.01). After intubation, excluding patients with cardiac arrest, 219 of 617 patients (35.5%) were given vasoactive medications.

## Discussion

This national survey provided comprehensive intubation characteristics for ED patients in Chinese EDs. The overall ED intubation process, including pretreatment, preoxygenation, induction, device selection, and any complications related to the intubation procedure were examined. This study, which covered more than half of the country's provinces, was the first large-scale cross-sectional survey of ED intubation in China. This study found some widespread issues related to ED intubation, and in this discussion section, we aim to review these and provide specific goals for improving ED intubation success rates in the near future. Although it may be unrealistic to hope that ED complication rates match those of the surgical OR, we can still aim to get as close as possible to maximize patient safety in the ED.

Regarding ED patients who were intubated in this study, the majority were elderly male patients with multiple chronic diseases who needed urgent intubation. Almost a quarter of all ED intubations involved patients with cardiac arrest. Unfortunately, Khandelwal et al. recently found that cardiac arrest was a predictor for difficult tracheal intubation (6). This may play a role in the relatively low first-pass success and high complication rates seen in this study.

Emergency department physicians alone performed the intubation procedure in most cases, and among ED physicians, most reported being proficient at intubation. There are different staff assignments in various hospitals, and it was intriguing to see that nurses performed 65 (6.4%) intubations in this study. We learned that eight out of 41 (19.5%) hospitals in this study had protocols for intubation performed by nurses. Examining why nurses are intubating and potentially including nurses in intubation training may be wise in the future. In addition, it is worth noting that about half of all intubations occurred during off-hours when senior doctors were unavailable, and most ED physicians performing intubations were ED residents. On the one hand, this phenomenon emphasizes the importance of early intubation training for junior ED physicians. On the other hand, allocating senior ED physicians to the period of high incidence could improve the quality of health care and decrease complications.

Regarding intubation device selection, video laryngoscopy was more popular than traditional direct laryngoscopy in Chinese EDs in this study. However, there is still some controversy over the best choice of equipment. A systematic review identified improved glottic view and minor airway trauma in the use of video laryngoscopy patients, but no differences in intubation numbers or complication rates (7). A recent meta-analysis demonstrated that video laryngoscopy is associated with reduced numbers of esophageal intubations compared to direct laryngoscopy during emergency intubation (8). However, Jean et al. found that video laryngoscopy was related to higher rates of severe adverse events among ICU patients (9). For beginners, however, the use of video laryngoscopy does appear to decrease rates of intubation failure (10).

As for induction drugs, we found usage rates of the three most common medications in China to be low compared to other countries (11). The emergent condition of many patients may contribute to this phenomenon to some extent. Among patients using sedatives, propofol and midazolam were most commonly selected. Etomidate, a drug with little effect on circulation, has proven to be effective in patients with circulatory instability (12), but its use in Chinese EDs is very rare. Although this study did not delve into the reasons for the lack of sedative and muscle relaxant medications being used in ED intubations, a lack of standardized training or a lack of medication in the ED may be the cause. Airway guidelines have recently been updated in the anesthesiology and emergency medicine fields (13), but, as is the case in many countries, clinical physicians are slow to change their practice.

It is well-known that preoxygenation plays a vital role in maintaining oxygen saturation during intubation. Some have recommended that all critically ill patients should be pre-oxygenated (14). Our data showed that measures to improve oxygenation were adopted in almost all (97.4%) patients peri-intubation, but the incidence of hypoxia was still high. Previous meta-analyses have identified noninvasive positive pressure ventilation as being superior to other preoxygenation measures prior to intubation (15, 16). Recently, the utility of apneic oxygenation in non-breathing patients has been confirmed (17, 18), and the application of high flow oxygen inhalation equipment has also increased. However, limited equipment and some patient characteristics (altered mental status, facial hair, etc.) made routine noninvasive positive pressure ventilation or high flow oxygenation difficult in the ED. Bag-valve mask ventilation was still the dominant traditional method of preoxygenation domestically and overseas (19). Nasal cannulas, which do not provide a high fraction of inspired oxygen, were used in about 20% of patients. Since a high pre-intubation oxygen concentration can delay the time until oxygen saturation declines (20), the more oxygen provided during preoxygenation, the better outcome. Finally, this study did not query the detailed process of how preoxygenation was performed. Future studies could focus on this point to explain the possible reasons behind the higher rates of hypoxia.

The Society for Airway Management in 2020 put forward consensus recommendations for airway management, including screening and pretreating patients for high risk of circulatory instability (21). However, in our investigation, among patients with hypotension (SBP <90 mmHg) before intubation, only 42% (175 of 422) received pretreatment. Neglecting pretreating circulatory problems in advance and incorrect use of induction agents may lead to more fluctuations in circulation. This was partly confirmed by a marked drop in blood pressure following intubation in this study. Overall, pretreatment before intubation and selecting proper induction medications are recommended areas for quality improvement in the future.

The first-pass intubation success rate has been associated with the incidence of adverse events during intubation (6) and is the most important statistic when it comes to intubations. Our results showed that 85.7% of patients were intubated successfully on the first attempt. This rate compares favorably to ED intubations in other countries, which range from 70.3 to 85% in several studies (2, 22–24). Unsurprisingly, physicians with greater intubation experience were associated with a higher intubation success rate. Unfortunately, using sedatives or muscle relaxants was associated with intubation failure in this study. This result is likely caused by selection bias or unmeasured confounders. One reason for the lower use of induction medications in Chinese EDs was that the most uncooperative or irritable patients were considered for these medications, which may have increased the difficulty of intubation. In addition, drug selection and timing may also affect intubation quality. Physicians may rush to establish an artificial airway rather than wait for medications to take effect. Although physicians may have knowledge of induction medications, the medications themselves are not being widely used. The reasons for this may include local hospital protocols, anesthesia limitations, or lack of real-world training.

After failing intubation for the first time, the top three rescue choices were changing intubators, attempting again, or changing devices. A “cannot intubate and cannot ventilate” scenario occurred in only one patient, who, following the wishes of his family, had care withdrawn. Finally, no supraglottic equipment was used in any patient in this study. Nevertheless, many Chinese EDs are equipped with supraglottic airway equipment and cricothyrotomy kits, but they seem to be rarely used.

We found a complication rate of 19.8% for ED intubations. This was a relatively high percentage compared to other national-level ED studies which found complication rates of 8–28% (6, 22, 25). Examining these studies, the proportion of different adverse events was not consistent. Walls listed the top three complications were esophageal intubation, mainstem bronchus intubation, and hypotension (26). Graham found that severe hypotension, esophageal intubation, and critical desaturation happened most often (27). In this study, the most frequent adverse event encountered was hypoxia, followed by hypotension and aspiration. The relatively higher rate of adverse events may be partly due to patients' underlying medical conditions, such as pulmonary disease and circulatory shock. Previous research has shown that lower initial oxygen saturation is an independent predictor of hypoxia (28, 29). Similarly, pre-intubation hypotension was correlated to post-intubation hypotension (30). In the ED, it may not be possible to raise oxygenation or SBP to “normal” levels prior to initiating the intubation procedure, but we can still try to maximize these factors as much as possible.

## Limitations

This study had some limitations. First, the survey only lasted 1 month, missing potential seasonal variations. Second, the number of hospitals chosen varied by region and some EDs enrolled only a small number of patients and may not represent a complete cross-section of all the intubations they performed. Third, this trial only focused on the short-term outcomes in the EDs. We still need further study into the long-term ED intubation outcomes, and a closer examination of controversial techniques, such as using cricoid pressure. Finally, this was a statistically descriptive cross-sectional study. The nature of the study is such that it can only uncover associations and cannot provide a further causal conclusion.

## Conclusion

This study illuminated the current circumstances surrounding intubations in Chinese EDs, including a relatively high adverse event rate. Nation-wide standardized ED intubation training should be enhanced, with an emphasis on induction agent education. Repeat surveys in 5–10 years will help see if there are significant changes to ED intubation adverse event and medication utilization rates.

## Data Availability Statement

The raw data supporting the conclusions of this article will be made available by the authors, without undue reservation.

## Ethics Statement

The studies involving human participants were reviewed and approved by Ethics Committee, Peking Union Medical College Hospital. The certificate number was JS-2718. The patients/participants provided their written informed consent to participate in this study.

## Author Contributions

JX and XY conceived and proposed the study. YD, HY, and JW were responsible for study design, statistical method selection, and drafting the manuscript. All authors reviewed and approved the final manuscript.

## Funding

This trial was supported by the CAMS (Chinese Academy of Medical Sciences) Innovation Fund for Medical Sciences (Project number 2017-I2M-1-009).

## Conflict of Interest

The authors declare that the research was conducted in the absence of any commercial or financial relationships that could be construed as a potential conflict of interest.

## Publisher's Note

All claims expressed in this article are solely those of the authors and do not necessarily represent those of their affiliated organizations, or those of the publisher, the editors and the reviewers. Any product that may be evaluated in this article, or claim that may be made by its manufacturer, is not guaranteed or endorsed by the publisher.
