# Supplementary material for: Tracheal Intubation in Emergency Departments in China: A National Cross-Sectional Survey
Source: Front Med (Lausanne). 2022 Feb 25;9:813833. doi: 10.3389/fmed.2022.813833 (PMC8914034; doi:10.3389/fmed.2022.813833)

Supplementary Material

**Tracheal Intubation in Emergency Departments in China: A National Cross-sectional Survey**

Yili Dai^1†^, Joseph Harold Walline^2†^, Heng Yu^3†^, Huadong Zhu^1^, Jun Xu^1^*, Xuezhong Yu^1^*

1 Emergency Department, State Key Laboratory of Complex Severe and Rare Diseases, Peking Union Medical College Hospital, Chinese Academy of Medical Science and Peking Union Medical College, Beijing, China.

2 Accident and Emergency Medicine Academic Unit, Chinese University of Hong Kong, Prince of Wales Hospital, No. 30-32 Ngan Shing Street, Shatin, NT, Hong Kong, China.

3 Emergency Department, Shapingba District People's Hospital, 44 Xiaolongkan New Street, Shapingba District, Chongqing, China.

*Correspondence:

Jun Xu, [Xujunfree@126.com](mailto:Xujunfree@126.com)

Xuezhong Yu, [yxz@pumch.cn](mailto:yxz@pumch.cn)

Figure S1. Flow chart of patients’ enrolling process


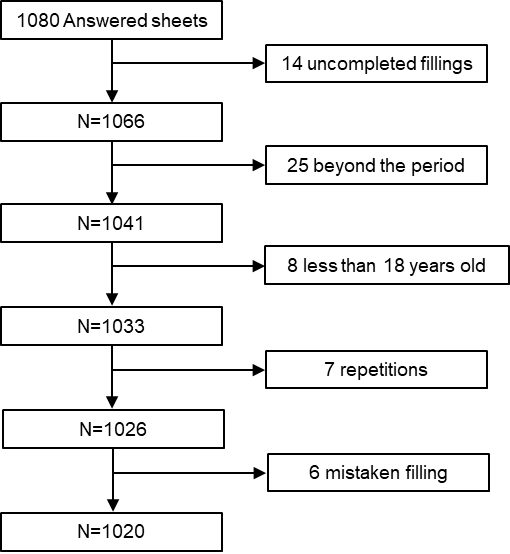

Supplement: Supplementary file 1 [file Data_Sheet_1.docx]
